# Is there a link between guttate psoriasis and SARS-CoV-2? A series of three cases^☆^

**DOI:** 10.1016/j.abd.2021.07.006

**Published:** 2021-12-30

**Authors:** Cláudia Brazão, Miguel Alpalhão, Marta Aguado-Lobo, Joana Antunes, Luís Soares-de-Almeida, Paulo Filipe

**Affiliations:** a Dermatology and Venereology Department, Hospital de Santa Maria, Centro Hospitalar Universitário Lisboa Norte, Lisbon, Portugal; b Dermatology and Venereology University Clinic, Faculty of Medicine, University of Lisbon, Lisbon, Portugal; c Dermatology Research Unit, iMM João Lobo Antunes, University of Lisbon, Lisbon, Portugal

Dear Editor,

Guttate psoriasis (GP) is an acute form of psoriasis that is associated with bacterial infections, mainly streptococcal, which cause superantigen-induced immune activation.^1^, ^2^ Viral upper respiratory infections may also be implicated, typically occurring two to three weeks before the onset of guttate lesions.^1^, ^3^

We report three cases of GP following Severe Acute Respiratory Syndrome Coronavirus 2 (SARS-CoV-2) infection and BNT162b2 mRNA vaccine.

A 42-year-old caucasian male with a history of chronic plaque psoriasis developed multiple erythematous scaly papules and plaques on the face, trunk, upper and lower limbs, and scaly erythematous plaques over the elbows and knees (Fig. 1), one week after he was diagnosed with Coronavirus Disease 2019 (COVID-19). We established a clinical and histopathological (Fig. 2) diagnosis of GP and a flare of plaque psoriasis.

**Figure 1:**
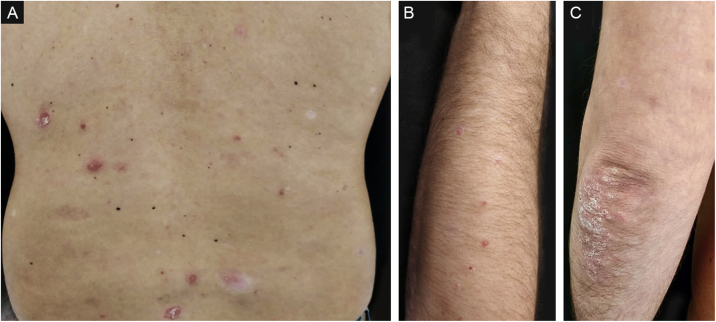
Guttate psoriasis and chronic plaque psoriasis flare (case 1): (A) Guttate psoriasis drop-like erythematous plaques and scaly erythematous plaques on the trunk; (B) Guttate psoriasis drop-like erythematous plaques on the upper limbs; (C) Scaly erythematous plaques on the elbows.

**Figure 2:**
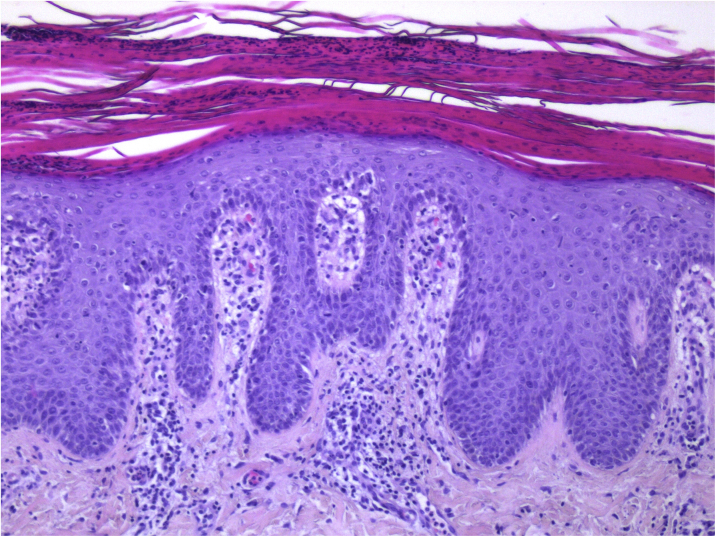
Guttate psoriasis histopathologic aspects (case 1) (Hematoxylin & eosin ×100): Psoriasiform hyperplasia of the epidermis, featuring neutrophils overlying continuous parakeratosis and intracorneal micro-abscesses; absence of stratum granulosum; papillomatosis with vascular hyperplasia and perivascular lymphocytic infiltrate in the superficial dermis.

A 32-year-old caucasian female with a history of chronic plaque psoriasis developed multiple erythematous scaly papules and plaques on the trunk, upper and lower limbs, as well as scaly erythematous plaques over the elbows and knees (Fig. 3), two weeks after she was diagnosed with COVID-19. We established a clinical diagnosis of GP and a flare of plaque psoriasis.

**Figure 3:**
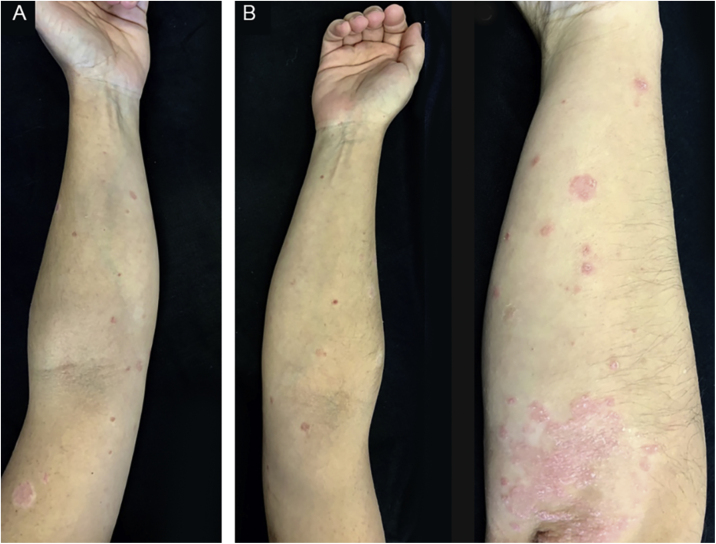
Guttate psoriasis and chronic plaque psoriasis flare (case 2): (A) Guttate psoriasis drop-like erythematous papules and plaques on the upper limbs; (B) Guttate psoriasis erythematous plaques on the upper limbs and erythematous scaly plaques on the elbows.

A 45-year-old caucasian male with a history of chronic plaque psoriasis presented with multiple erythematous scaly papules and small plaques on the trunk and upper limbs, one week following the first dose of COVID-19 BNT162b2 mRNA vaccine, with worsening after the second dose. A clinical diagnosis of GP was made.

The correlation between psoriasis and infection is well established, and viruses are recognized triggers. In one study of viral respiratory infections causing psoriasis flares, coronavirus was one of the most frequently detected pathogens.^3^

SARS-CoV-2 spike (S) protein exhibits a high-affinity motif for T-Cell Receptors (TCR) and may form a ternary complex with Major Histocompatibility Complex type 2 (MHC-II). Its binding epitope harbors a sequence motif that is very similar in sequence and structure to bacterial superantigens.^4^ Therefore, SARS-CoV-2 may favor psoriasis through superantigen modulation of the adaptive immune response. In fact, analysis of COVID-19 patients demonstrates that they exhibit TCR arrangements consistent with superantigen activation.^4^ S protein may cause activation and polyclonal expansion of T-cells in the upper respiratory tract (namely the tonsils), causing them to differentiate and migrate to cutaneous tissue after acquiring skin-homing capacity through increased expression of Cutaneous Lymphocyte Antigen (CLA).^2^, ^4^ These mechanisms may contribute to the inflammatory imbalance that underlies the pathophysiology of psoriasis, particularly acute forms such as GP.

BNT162b2 COVID-19 vaccine consists of nucleoside-modified mRNA encoding the full-length spike protein, which will be massively produced and expressed on the surface of host cells, mimicking the structure and expression of the wild-type S protein during natural infection.^5^ Thus, it is expected that the host immune responses to the vaccine will be similar to those during natural infection, explaining, through the abovementioned mechanism, how this vaccine could lead to acute forms of psoriasis in the absence of actual infection.

These clinical cases demonstrate the importance of cutaneous abnormalities in patients with COVID-19, as well as possible dermatologic events in patients undergoing vaccination, and shed light on the potential immunologic underlying mechanisms.

## Financial support

None declared.

## Authors’ contributions

Cláudia Brazão: Approval of the final version of the manuscript; critical literature review; data collection, analysis, and interpretation; effective participation in research orientation; intellectual participation in propaedeutic and/or therapeutic management of studied cases; critical manuscript review; preparation and writing of the manuscript; study conception and planning.

Miguel Alpalhão: Approval of the final version of the manuscript; critical literature review; data collection, analysis, and interpretation; effective participation in research orientation; intellectual participation in propaedeutic and/or therapeutic management of studied cases; critical manuscript review; preparation and writing of the manuscript; study conception and planning.

Marta Aguado-Lobo: Approval of the final version of the manuscript; critical literature review; data collection, analysis, and interpretation; effective participation in research orientation; intellectual participation in propaedeutic and/or therapeutic management of studied cases; critical manuscript review; preparation and writing of the manuscript.

Joana Antunes: Approval of the final version of the manuscript; critical literature review; effective participation in research orientation; intellectual participation in propaedeutic and/or therapeutic management of studied cases; critical manuscript review; preparation and writing of the manuscript; study conception and planning.

Luís Soares-de-Almeida: Approval of the final version of the manuscript; critical literature review; effective participation in research orientation; intellectual participation in propaedeutic and/or therapeutic management of studied cases; critical manuscript review; preparation and writing of the manuscript; study conception and planning.

Paulo Filipe: Approval of the final version of the manuscript; Critical literature review; Data collection, analysis, and interpretation; Effective participation in research orientation; Intellectual participation in propaedeutic and/or therapeutic management of studied cases; Manuscript critical review; preparation and writing of the manuscript; Study conception and planning.

## Conflicts of interest

None declared.
